# Epidemiology of Spinocerebellar Ataxias in Europe

**DOI:** 10.1007/s12311-023-01600-x

**Published:** 2023-09-12

**Authors:** Filippo De Mattei, Fabio Ferrandes, Salvatore Gallone, Antonio Canosa, Andrea Calvo, Adriano Chiò, Rosario Vasta

**Affiliations:** 1 ALS Center, Department of Neuroscience “Rita Levi Montalcini”, University of Turin, Turin, Italy; 2 Aging Brain and Memory Clinic, Department of Neuroscience “Rita Levi Montalcini”, University of Turin, Turin, Italy; 3 Neurology 1, AOU Città Della Salute E Della Scienza Di Torino, Turin, Italy; 4 Institute of Cognitive Science and Technologies, National Research Council, Rome, Italy

**Keywords:** Autosomal dominant cerebellar ataxias, Spinocerebellar ataxias, SCA, Epidemiology, Review

## Abstract

Spinocerebellar ataxias (SCAs) are a heterogenous group of rare neurodegenerative conditions sharing an autosomal dominant pattern of inheritance. More than 40 SCAs have been genetically determined. However, a systematic review of SCA epidemiology in Europe is still missing. Here we performed a narrative review of the literature on the epidemiology of the most common SCAs in Europe. PubMed, Embase, and MEDLINE were searched from inception until 1 April 2023. All English peer-reviewed articles published were considered and then filtered by abstract examination and subsequently by full text reading. A total of 917 original articles were retrieved. According to the inclusion criteria and after reviewing references for useful papers, a total of 35 articles were included in the review. Overall, SCA3 is the most frequent spinocerebellar ataxia in Europe. Its frequency is strikingly higher in Portugal, followed by Germany, France, and Netherlands. None or few cases were described in Italy, Russia, Poland, Serbia, Finland, and Norway. SCA1 and SCA2 globally displayed similar frequencies, and are more prevalent in Italy, United Kingdom, Poland, Serbia, and France.

**Supplementary Information:**

The online version contains supplementary material available at 10.1007/s12311-023-01600-x.

## Introduction

Spinocerebellar ataxias (SCAs) or autosomal dominant ataxias (ADCAs) are a heterogeneous group of genetic disorders characterized by the degeneration of the cerebellum and its connected structures. Consistently, SCA phenotypes encompass progressive cerebellar and non-cerebellar symptoms [1, 2]. In 1891, Menzel first reported the case of a 28-year-old patient with a familiar progressive cerebellar ataxia. Since that first description, many case reports have been published. In 1983, Anita Harding distinguished three phenotypes: ADCA type I, characterized by progressive cerebellar ataxia associated with extracerebellar signs and symptoms, with the main pathological finding being olivopontocerebellar atrophy; ADCA type II characterized by cerebellar ataxia and retinal degeneration; and ADCA type III, which involved relatively isolated cerebellar ataxia [3]. More recently, thanks to the advancement in genetic diagnostic technologies, over 40 clinical entities have been recognized [4]. It is now known that the global prevalence of SCAs ranges from 0 to 5.6 cases per 100,000 persons [1, 5].

Here our aim was to conduct a narrative review of the literature focusing on the epidemiology of SCAs and their distribution in Europe.

## Materials and Methods

We performed a narrative review of peer-reviewed articles in full and only in English. The search was conducted in PubMed, MEDLINE, and Embase using the string extensively reported in supplementary materials as of April 1, 2023. The research results were filtered by “full text” availability. The review design was not restricted by study design. The eligible criteria were first evaluated by abstract examination and subsequently by full text reading. References of the collected published studies were also considered. The remaining articles were then screened including only those focusing on (a) SCA1, SCA2, SCA3, SCA6, SCA7, SCA8, and SCA17; (b) European countries; and (c) epidemiological data such as prevalence, incidence, and relative frequency (RF, namely, the ratio between frequency of each specific SCA and the overall frequency of all SCAs detected).

## Results

**Fig. 1:**
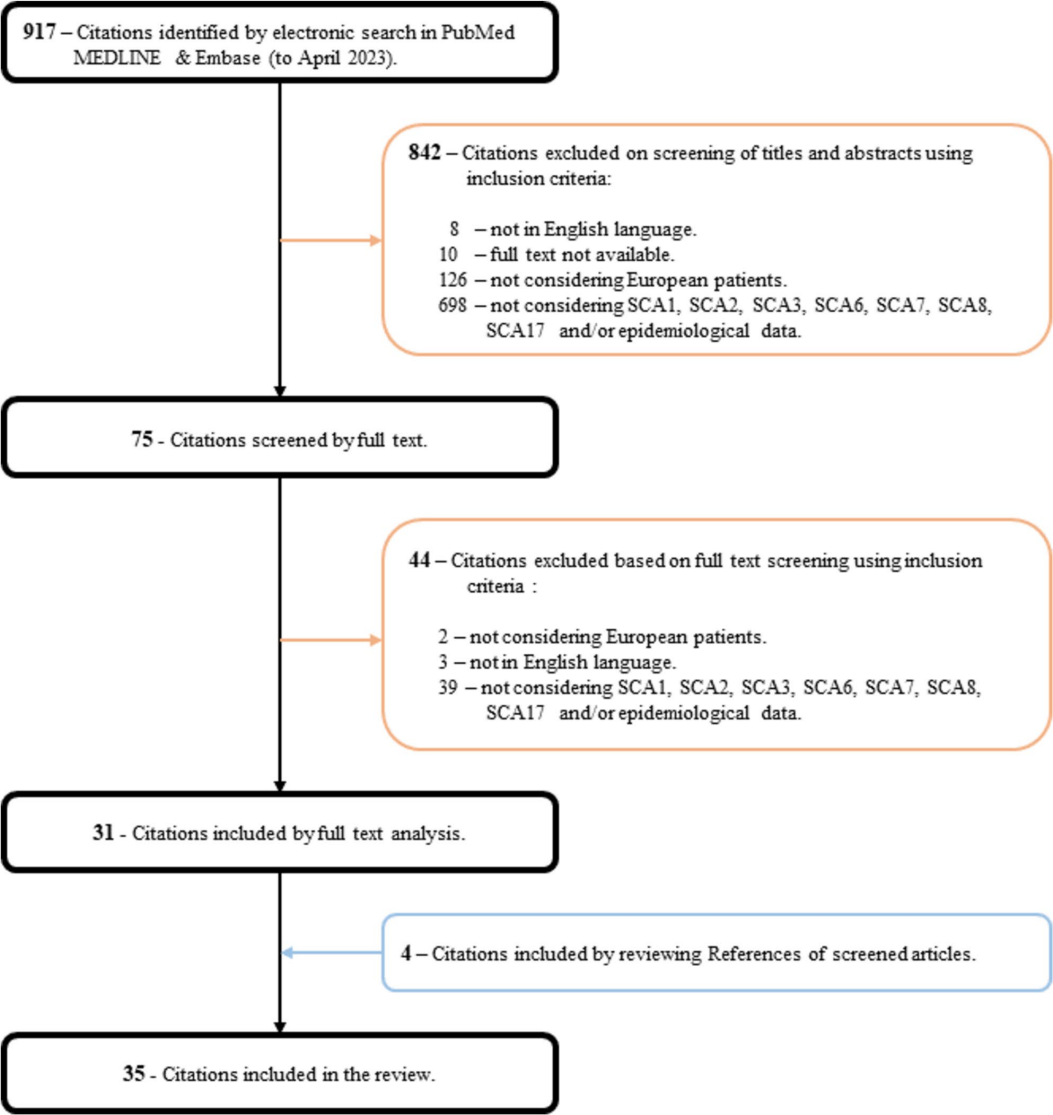
Flowchart showing number of citations retrieved by database searching and the number and type of studies included in this review

Through the electronic searching, we retrieved a total of 917 original articles. After screening the abstracts, we excluded 842 articles that did not meet the inclusion criteria. Among the remaining articles, 44 were further excluded after reading their full text. The process of reviewing references allowed the inclusion of 4 additional articles. Ultimately, a total of 35 articles were included in the review. A visual representation of the selection phases is illustrated in Fig. 1.

### SCA1

SCA1 was the first ADCA to be genetically characterized [6]. It is caused by the expansion of CAG triplet repeats in the N-terminal coding region of the *ATXN1* gene. The mutation leads to a polyglutamine expansion in the encoded protein, ataxin-1, that ultimately causes the loss of cerebellar Purkinje cells as long as of neurons of the dentate, basal, pontine, and olivary nuclei [7]. The typical onset of SCA1 occurs in the fourth to fifth decade of life. However, similar to other diseases caused by triplet repeats, the age of onset and the severity of the phenotype are inversely related to the number of repeats present [8]. Patients show a combination of cerebellar signs (ataxia, slow saccades, ophthalmoplegia), pyramidal signs (hyperreflexia, extensor plantar sign, urinary dysfunction), and peripheral neuropathy [9]. Cognitive impairment, peripheral signs (muscle atrophy, also facio-lingual, associated with fasciculations), optic atrophy, and extrapyramidal signs (dystonia, myoclonus, rigidity, resting tremor, chorea) can be observed albeit less commonly [10–13]. The prevalence and the RF of SCA1 vary significantly across countries. In Europe, SCA1 and SCA2 have similar RF of approximately 25% and are generally less common compared to SCA3 [8, 14].

Notably, two Polish studies reported the highest RF of SCA1 among European countries, with percentages of 42% and 68%, respectively [15, 16]. Specifically, in a study including 203 unrelated patients with inherited ataxias, 168 (68%) tested positive for SCA1 expansions. The estimated prevalence of SCA1 was therefore 1 in 100,000 individuals. This data has been attributed to a founder effect, as 61 patients originated from the Mazowieckie voivodeship in the middle of Poland, where the prevalence was 1 in 41,301. Among these patients, 41 probands carried the same 197-bp variant [16].

**Fig. 2:**
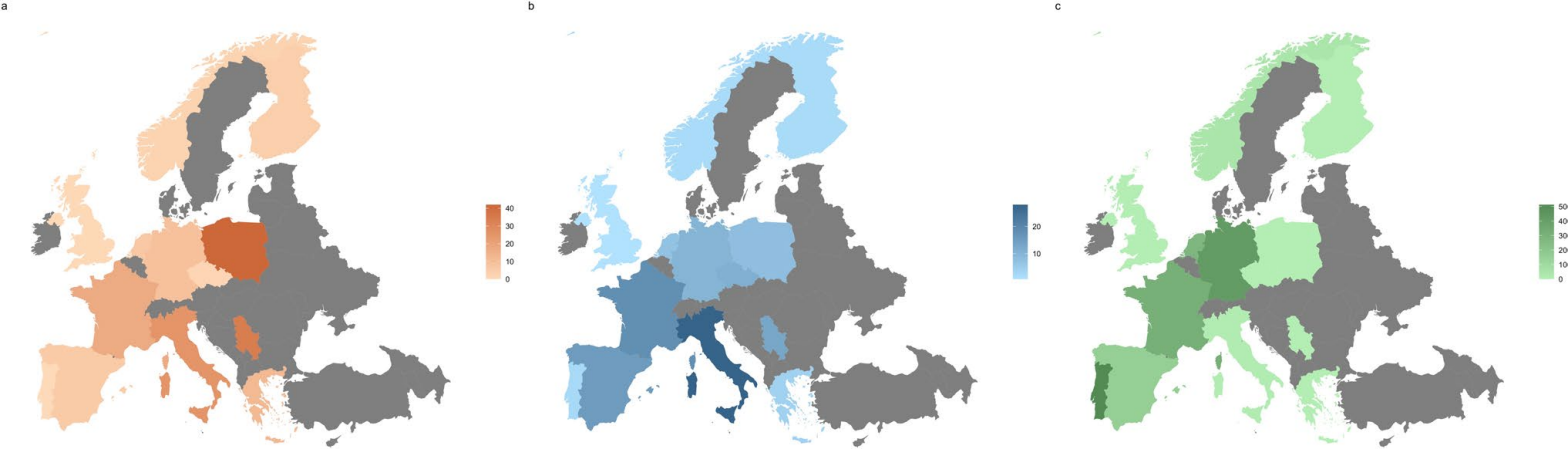
Europe map showing the relative frequency of SCA1 (**a**), SCA2 (**b**), and SCA3 (**c**) according to the studies retrieved. Gray refers to countries for which SCA epidemiology is missing. Countries for which multiple studies were available (namely, Finland, France, Italy, Norway, Poland, Portugal, Spain, and United Kingdom) were represented based on the estimates from the study with the highest number of individuals/families included. Iceland, Svalbard Islands, Russia, and Kazakhstan were not included for the sake of image’s dimensions

In Serbia, SCA1 was diagnosed in 13 out of 38 families affected by dominant cerebellar ataxia, resulting in a RF of 34%. Also in this case, 9 families originated from a small, south-eastern region of Serbia but no data were provided about the heterogeneity of mutations [17]. Similarly, SCA1 mutations were found in 5 out of 15 Russian families with dominant cerebellar ataxia, indicating an RF of 33% [18]. In Italy, three major studies conducted nationwide reported RF values for SCA1 ranging from 41 to 25.3% and 24% [19–21]. However, the prevalence of SCA1 varies significantly across regions within Italy. According to the geographic origin of the probands, Northern Italy had the highest RF for SCA1 at 72%, while Southern Italy showed the highest frequency for SCA2 at 63% [19, 21]. In central Italy, a study focused on patients referred to neurological centers in Perugia and Florence found SCA1 to be the second most common subtype with an RF of 19% [22]. However, this does not seem to be a rule and different frequencies in specific locations have been described. A study conducted in the province of Padua, Northeast Italy, reported a prevalence ratio of 13 per 1,000,000 population and an RF of 13.9%, similar to SCA2 [11]. Another study found a striking RF of 91.6% for SCA1 among probands from mid-eastern Sicily, despite the sample size was limited [23]. Greece also showed a predominant RF of 12% for SCA1, which was shared with SCA7 [24]. In France (14.1–17.4%) [25–28], Germany (9%) [13], Finland (4%) [29], the Netherlands (6.2%) [30], and Portugal (2.2%) [31] SCA1 had similar frequencies to SCA2 and lower frequencies than SCA3. In all other countries, SCA1 had a lower RF among other dominant cerebellar ataxias, with values ranging from 0 to 2% (United Kingdom [32–34], Czech Republic [35], and Norway [36]) to approximately 5% (Spain [37, 38]) (Table 1 and Fig. 2a).

**Table 1:**
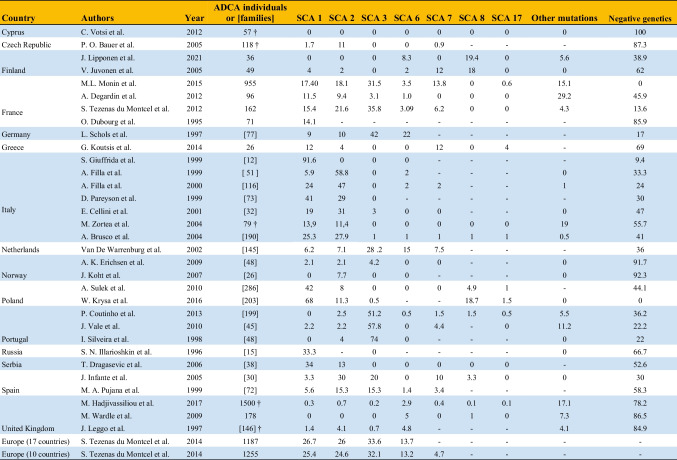
Relative frequencies (%) of SCA1, SCA2, SCA3, SCA6, SCA7, SCA8, and SCA17 among autosomal dominant cerebellar ataxia (ADCA) families or individuals across different European countries

Negative genetics refers to patients for whom the analyses of genes known at the time of the study were negative. The “†” symbol refers to studies also including patients affected by autosomal recessive and sporadic cerebellar ataxias

### SCA2

SCA2 is caused by the CAG repeat expansions in the *ATXN2* gene. The mutation results in the polyglutamine expansion in the ataxin-2 protein that ultimately causes the loss of Purkinje cells [39]. Along with cerebellar signs, SCA2 patients could also show pyramidal signs (spasticity, urinary dysfunction, extensor plantar, hyperreflexia), peripheral nerve signs (muscular atrophy, areflexia, fasciculations), cognitive impairment, and extrapyramidal signs (resting tremor, myoclonus, dystonia, rigidity, dyskinesia, and choreic movements) [10, 13, 19, 37]. According to the European integrated project on spinocerebellar ataxias register (EUROSCA), SCA2 is overall less common than SCA3 and has a frequency comparable to SCA1 in European countries [8, 14]. However, its frequency is not homogenous across countries.

In Italy, SCA2 appears to be the most reported ADCA, accounting for up to 27.9% of cases identified within 190 ADCA families [21]. As previously noted for SCA1, discrepancies arise when comparing different subregions of Italy, with the highest RF reported in Southern Italy (58.8%) [40] and the lowest in the Northeast (11.4%) [11].

In Spain, Pujana et al. analyzed a large sample of 72 ADCA families from 9 different autonomous communities and reported a RF of 15.3% for SCA2, similar to that of SCA3 [38]. However, with a smaller sample size and a more geographically homogeneous population, Infante et al. found a higher RF of 30% in the northern part of the country, where SCA2 frequency surpasses the RF of SCA3 [37].

In France, SCA2 was the second most frequent type of SCA in all included studies, with RFs ranging from 9.4 to 21.6% [25–28]. Although SCA3 is the most frequently reported subtype in the country overall, when analyzing patients from the northern part of France specifically, SCA1 appears to be the most prevalent [26].

Eastern European countries, such as Serbia (RF 13%) [17], Poland (RF 11.3%) [16], and Czech Republic (RF 11%) [35], also exhibit a relatively high prevalence of SCA2. In Czech Republic SCA2 is the most frequent SCA subtype reported [35]. In all other countries, SCA2 is less represented, with RFs ranging from 2 to 10% (Table 1 and Fig. 2b).

### SCA3

SCA3, also known as Machado-Joseph Disease (MJD), was initially referred to as the “Azorean disease of the nervous system” due to its first description in 1977 within three Portuguese-Azorean families [2, 33]. Since then, SCA3 has been reported in numerous families worldwide, both Portuguese and non-Portuguese [34]. The disease is caused by the expansion of CAG triplets in the ataxin-3 gene (*ATXN3*) [1]. Based on clinical presentation and age at onset, MJD can be further classified into three subtypes: (i) Type I or “Joseph,” characterized by early onset, cerebellar ataxia, pyramidal, and extrapyramidal signs; (ii) Type II or “Thomas,” associated with cerebellar ataxia, pyramidal signs, ophthalmoplegia, and an intermediate age of onset; and (iii) Type III or “Machado,” characterized by peripheral neuropathy and later onset age [33]. Along with cerebellar signs such as ataxia, saccadic smooth pursuit, gaze-evoked nystagmus, dysarthria, and dysphagia, patients with SCA3 also exhibit pyramidal signs and peripheral neuropathy. Extrapyramidal signs and cognitive impairment are less frequently described [13].

SCA3 is the most common dominant cerebellar ataxia in Europe, with a RF of 32–33% [8, 14]. Its highest prevalence is found among inhabitants of the Azores Islands and Portugal. In a late 90 s study involving 46 Portuguese families with dominant cerebellar ataxia, 74% were affected by SCA3, with half of those families originating from the Azores Islands [41]. A similar high prevalence was observed in the Tagus River Valley, on the Portuguese mainland, where SCA3 is estimated to affect 1 in 1000 inhabitants [2]. More recent studies published in 2010 and 2013, with the latter having a larger sample size of 199 ADCA Portuguese families, confirmed SCA3 as the most common SCA subtype in Portugal, with RFs of 57.4% and 51.2%, respectively [31, 42].

Outside of Portugal, SCA3 has the highest RF among SCAs only in Germany [13], the Netherlands [30], and France [25–27], accounting for 42%, 28.2%, and 30–35% of patients with ADCA, respectively. In Spain, SCA2 and SCA3 have similar RF of approximately 15% [38]. In all other countries, the RF of SCA3 ranges from 0 to 5% and is the least common dominant cerebellar ataxia (Table 1 and Fig. 2c).

### Other Less Common SCAs

SCA6 is caused by an expansion of the CAG trinucleotide repeat in the *CACNA1A* gene, which encodes for a calcium channel subunit. SCA6 usually onset in mid-adulthood and is characterized by slowly progressive ataxia, nystagmus, and dysarthria. Less commonly, patients can also present migraine headache, episodic ataxia, and other neurological symptoms [43]. According to the EUROSCA register, SCA6 has an overall RF of 13% in European countries [8, 14]. However, Germany [13] and the Netherlands [30] show higher RF values of 22% and 15%, respectively. In the United Kingdom, SCA6 has a lower RF (from 2.9 to 5%), which may be due to the inclusion in the study of sporadic cases or patients with a family history of autosomal recessive ataxia alongside ADCA [32–34]. Despite the lower RF, SCA6 is the most frequent diagnosed subtype overall in the United Kingdom. It is worth noting that the majority of European countries included in the studies reported either no or few cases of SCA6 (Table 1).

SCA7 is caused by an expansion of the CAG repeat in the *ATXN7* gene, which encodes for the ataxin-7 protein. This subtype is rare and typically presents in early adulthood with a combination of cerebellar ataxia, retinal degeneration, and pyramidal signs. SCA7 has a high penetrance, meaning that almost all individuals with the expansion will develop symptoms [44]. The EUROSCA register data show a RF of 4.7% for SCA7 [14]. Finland [29], France [25], Greece [24], the Netherlands [30], and Spain [37] reported the highest RF values ranging from 7.5 to 13.8%. Other countries had either no or few detected cases, with RF values below 4.4% (Table 1).

 SCA8 is caused by CTG/CAG trinucleotide repeat expansion in the ATXN8 gene, putatively leading to RNA-mediated neurotoxicity. This subtype is typically characterized by slowly progressive ataxia and cerebellar dysarthria typically occurring in mid-life [45]. Finland [29, 46] shows remarkable RF values for SCA8, ranging from 18 to 19.4%, while Poland [15, 16] reports values of 4.9 to 18.7%. However, in most European countries, SCA8 has either not been investigated or is scarcely represented, with RF values below 3.3% (Table 1).

Finally, SCA17, also known as Huntington disease-like 4 (HDL4), is caused by an expansion of the TATA box-binding protein (*TBP*) gene, which encodes for a protein involved in gene transcription. This subtype is rare and exhibits a wide range of clinical features, including ataxia, dementia, chorea, and psychiatric symptoms. The age of onset and severity of symptoms can vary widely, even among individuals with the same expansion [47]. The only study reporting a relevant RF value is from Greece [24], with an RF of 4%. Similar to SCA8, SCA17 has been rarely investigated and poorly retrieved in the diagnostic panel of other countries, with RF values below 1.5% (Table 1).

## Discussion

As expected for genetic Mendelian diseases, the epidemiology of SCAs is extremely heterogeneous across European countries, and even within different subregions of the same country. Overall, SCA3 is the most frequent ADCA subtype in Europe. However, its prevalence remains high among families in Portugal, Germany, France, the Netherlands, and Spain, while in other countries none or few cases were reported. SCA1 and SCA2 are more diffuse across Europe, showed similar estimates, and were more frequent in Italy, France, Serbia, and Poland. Unlike SCA3 and other SCA subtypes considered, very few studies have found no SCA1 or SCA2 cases among ADCA families.

As for less common SCA subtypes, although these are generally poorly represented overall, a few clusters have been identified in specific countries such as Finland (SCA6, SCA7, SCA8), France (SCA7), Germany (SCA7), Greece (SCA7), Netherlands (SCA6, SCA7), and Poland (SCA8).

The differences in the distribution of SCAs across different countries are likely the result of population migrations coupled with founder effects in isolated geographic areas [48, 49]. This explains the higher frequencies reported within specific small subregions, such as the Flores Islands (an island of the western group of the Azores) in Portugal for SCA3 and the Mazowieckie voivodeship in Poland for SCA1.

The presence of a significant percentage of genetically unidentified disorders in ADCA families highlights the necessity of further improvements in diagnostic techniques and a deeper understanding of the underlying pathophysiology.

In conclusion, the frequencies of SCA1, SCA2, SCA3, SCA6, SCA7, SCA8, and SCA17 vary significantly between countries. When genetic analyses are carried out in clinical settings and family mutations are unknown, it is crucial to consider local epidemiological reports along with neurological findings. There is a need for further studies to investigate the epidemiology of less common subtypes of SCAs.

### Supplementary Information

Below is the link to the electronic supplementary material.

Supplementary file1 (DOCX 25 KB)

## Data Availability

Data sharing is not applicable to this article as no new data were created or analyzed in the study.
