# Supplementary material for: Epidemiology of Spinocerebellar Ataxias in Europe
Source: Cerebellum. 2023 Sep 12;23(3):1176–83. doi: 10.1007/s12311-023-01600-x (PMC11102384; doi:10.1007/s12311-023-01600-x)
Supplement: Supplementary file 1 — Supplementary file1 (DOCX 25 KB) [file 12311_2023_1600_MOESM1_ESM.docx]

# **Supplementary material 1A. Search string used in Pubmed for the literature research.**

("dominant cerebellar ataxia*"[title/abstract] OR ADCA[title/abstract] OR ADCAs[title/abstract] OR "spinocerebellar ataxia 1"[title/abstract] OR "spino-cerebellar ataxia 1"[title/abstract] OR SCA1[title/abstract] OR "spinocerebellar ataxia 2"[title/abstract] OR "spino-cerebellar ataxia 2"[title/abstract] OR SCA2[title/abstract] OR "Machado-Joseph Disease"[Mesh] OR "spinocerebellar ataxia 3"[title/abstract] OR "spino-cerebellar ataxia 3"[title/abstract] OR SCA3[title/abstract] OR "Machado-Joseph"[title/abstract] OR "Joseph disease"[title/abstract] OR "Machado disease"[title/abstract] OR "Machado syndrome"[title/abstract] OR "Azorean disease"[title/abstract] OR "Azorean neurologic disease"[title/abstract] OR "Azorean ataxia"[tiab] OR "nigrospinodentatal degeneration*"[title/abstract] OR "nigro-spino-dentatal degeneration*"[title/abstract] OR "spinocerebellar ataxia 6"[title/abstract] OR "spino-cerebellar ataxia 6"[title/abstract] OR SCA6[title/abstract] OR "spinocerebellar ataxia 7"[title/abstract] OR "spino-cerebellar ataxia 7"[title/abstract] OR SCA7[title/abstract] OR ("pigmentary retinopath*"[title/abstract] OR "pigmentary maculopath*"[title/abstract] OR "retinal degenerat*"[title/abstract] AND ataxi*[title/abstract]) OR "Spinocerebellar ataxia 8"[Supplementary Concept] OR "spinocerebellar ataxia 8"[title/abstract] OR "spino-cerebellar ataxia 8"[title/abstract] OR SCA8[title/abstract] OR "Spinocerebellar Ataxia 17"[Supplementary Concept] OR "spinocerebellar ataxia 17"[title/abstract] OR "spino-cerebellar ataxia 17"[title/abstract] OR SCA17[title/abstract] OR "Huntington Disease-Like 4"[title/abstract] OR HDL4[title/abstract] OR (("Spinocerebellar Degenerations"[Mesh:noexp] OR "Spinocerebellar Ataxias"[Mesh] OR "spinocerebellar ataxia*"[title/abstract] OR "spinocerebellar degeneration*"[title/abstract] OR "spinocerebellar disease*"[title/abstract] OR "spino-cerebellar ataxia*"[title/abstract] OR "spino-cerebellar degeneration*"[title/abstract] OR "spino-cerebellar disease*"[title/abstract]) AND ("type 1"[title/abstract] OR "type 2"[title/abstract] OR "type 3"[title/abstract] OR "type 6"[title/abstract] OR "type 7"[title/abstract] OR "type 8"[title/abstract] OR "type 9"[title/abstract] OR "type I"[title/abstract] OR "type II"[title/abstract] OR "type III"[title/abstract] OR "type VI"[title/abstract] OR "type VII"[title/abstract] OR "type VIII"[title/abstract] OR "type IX"[title/abstract])) OR (("Ataxin-1"[Mesh] OR "ataxin-1"[title/abstract] OR "atxn-1"[title/abstract] OR atxn1[title/abstract] OR "Ataxin-2"[Mesh] OR "ataxin-2"[title/abstract] OR "atxn-2"[title/abstract] OR atxn2[title/abstract] OR "atx-2"[title/abstract] OR "Ataxin-3"[Mesh] OR "ataxin-3"[title/abstract] OR "atxn-3"[title/abstract] OR atxn3[title/abstract] OR CACNA1A[title/abstract] OR "Ataxin-7"[Mesh] OR "ataxin-7"[title/abstract] OR "atxn-7"[title/abstract] OR atxn7[title/abstract] OR "ATXN8 protein, human"[Supplementary Concept] OR "ataxin-8"[title/abstract] OR "atxn-8"[title/abstract] OR atxn8[title/abstract] OR "TATA Box Binding Protein-Like Proteins"[Mesh] OR "TATA Box”[title/abstract] OR "TBP gene"[title/abstract]) AND ("Cerebellar Ataxia"[Mesh] OR "Spinocerebellar Degenerations"[Mesh] OR ataxia*[title/abstract] OR cerebell*[title/abstract] OR spinocerebell*[title/abstract])))

AND

("Global Burden of Disease"[Mesh] OR "Global Health"[Mesh] OR global[title/abstract] OR worldwide[title/abstract] OR world-wide[title/abstract] OR "Europe"[Mesh] OR "European People"[Mesh] OR Europe[title/abstract] OR European[title/abstract] OR Europeans[title/abstract] OR "Alpine region*"[title/abstract] OR "Alpine area*"[title/abstract] OR "Alpine countr*"[title/abstract] OR "Mediterranean region*"[title/abstract] OR "Mediterranean area*"[title/abstract] OR "Mediterranean countr*"[title/abstract] OR Baltic[title/abstract] OR Baltics[title/abstract] OR Balts[title/abstract] OR Estonia[title/abstract] OR Estonian[title/abstract] OR Estonians[title/abstract] OR Tallin[title/abstract] OR Latvia[title/abstract] OR Latvian[title/abstract] OR Latvians[title/abstract] OR Letts[title/abstract] OR Riga[title/abstract] OR Courland[title/abstract] OR Curonian[title/abstract] OR Curonians[title/abstract] OR Lithuania[title/abstract] OR Lithuanian[title/abstract] OR Lithuanians[title/abstract] OR Vilnius[title/abstract] OR Russia[title/abstract] OR Russian[title/abstract] OR Russians[title/abstract] OR Moskow[title/abstract] OR Bashkiria[title/abstract] OR Bashkirian[title/abstract] OR Bashkirians[title/abstract] OR Bashkortostan[title/abstract] OR Dagestan[title/abstract] OR Dagestani[title/abstract] OR Dagestanian[title/abstract] OR Dagestanians[title/abstract] OR Daghestan[title/abstract] OR Daghestani[title/abstract] OR Daghestanian[title/abstract] OR Daghestanians[title/abstract] OR Tatar[title/abstract] OR Tatars[title/abstract] OR Siberia[title/abstract] OR Siberian[title/abstract] OR Siberians[title/abstract] OR Karelia[title/abstract] OR Karelian[title/abstract] OR Karelians[title/abstract] OR Carelia[title/abstract] OR Carelian[title/abstract] OR Carelians[title/abstract] OR Crimea[title/abstract] OR Crimean[title/abstract] OR Crimeans[title/abstract] OR Armenia[title/abstract] OR Armenian[title/abstract] OR Armenians[title/abstract] OR Transcaucasia[title/abstract] OR Transcaucasian[title/abstract] OR Transcaucasians[title/abstract] OR Azerbaijan[title/abstract] OR Azerbaijani[title/abstract] OR Azerbaijanis[title/abstract] OR Nagorno-Karabakh[title/abstract] OR Artsakh*[title/abstract] OR "Georgian Republic"[title/abstract] OR Abkhasia[title/abstract] OR Abkhasian[title/abstract] OR Abkhasians[title/abstract] OR Ossetia[title/abstract] OR Ossetian[title/abstract] OR Ossetians[title/abstract] OR Belarus[title/abstract] OR Belarussian[title/abstract] OR Belarussians[title/abstract] OR Byelorussia[title/abstract] OR Byelorussian[title/abstract] OR Byelorussians[title/abstract] OR Belorussia[title/abstract] OR Belorussian[title/abstract] OR Belorussians[title/abstract] OR Byelarus[title/abstract] OR Byelarussian[title/abstract] OR Byelarussians[title/abstract] OR Belorussia[title/abstract] OR Belorussian[title/abstract] OR Belorussians[title/abstract] OR Minsk[title/abstract] OR Ukraine[title/abstract] OR Ukrainian[title/abstract] OR Ukrainians[title/abstract] OR Kiev[title/abstract] OR Bessarabia[title/abstract] OR Bessarabian[title/abstract] OR Bessarabians[title/abstract] OR Bukovina[title/abstract] OR Bukovinian[title/abstract] OR Bukovinians[title/abstract] OR Crimea[title/abstract] OR Crimean[title/abstract] OR Crimeans[title/abstract] OR Moldova[title/abstract] OR Moldovan[title/abstract] OR Moldovans[title/abstract] OR Moldavia[title/abstract] OR Moldavian[title/abstract] OR Moldavians[title/abstract] OR Chisinau[title/abstract] OR Poland[title/abstract] OR Polish[title/abstract] OR Polska[title/abstract] OR Warsaw[title/abstract] OR Warszaw[title/abstract] OR Lemko[title/abstract] OR Lemkos[title/abstract] OR Lemkian[title/abstract] OR Lemkians[title/abstract] OR Lemk[title/abstract] OR Lemks[title/abstract] OR Pomerania[title/abstract] OR Pomeranian[title/abstract] OR Pomeranians[title/abstract] OR Silesia[title/abstract] OR Silesian[title/abstract] OR Silesians[title/abstract] OR Moravia[title/abstract] OR Moravian[title/abstract] OR Moravians[title/abstract] OR Bulgaria[title/abstract] OR Bulgarian[title/abstract] OR Bulgarians[title/abstract] OR Sofia[title/abstract] OR Thrace[title/abstract] OR Thracian[title/abstract] OR Thracians[title/abstract] OR Romania[title/abstract] OR Romanian[title/abstract] OR Romanians[title/abstract] OR Rumania[title/abstract] OR Rumanian[title/abstract] OR Rumanians[title/abstract] OR Bucharest[title/abstract] OR Bucuresti[title/abstract] OR Transylvania[title/abstract] OR Transylvanian[title/abstract] OR Transylvanians[title/abstract] OR Transilvania[title/abstract] OR Transilvanian[title/abstract] OR Transilvanians[title/abstract] OR Wallachia[title/abstract] OR Wallachian[title/abstract] OR Wallachians[title/abstract] OR Hungary[title/abstract] OR Hungarian[title/abstract] OR Hungarians[title/abstract] OR Magyar[title/abstract] OR Magyars[title/abstract] OR Budapest[title/abstract] OR Swabia[title/abstract] OR Swabian[title/abstract] OR Swabians[title/abstract] OR Slovakia[title/abstract] OR Slovak[title/abstract] OR Slovaks[title/abstract] OR Slovakian[title/abstract] OR Slovakians[title/abstract] OR Bratislava[title/abstract] OR Czech[title/abstract] OR Czechia[title/abstract] OR Czechs[title/abstract] OR Bohemia[title/abstract] OR Bohemian[title/abstract] OR Bohemians[title/abstract] OR Prague[title/abstract] OR Slovenia[title/abstract] OR Slovenian[title/abstract] OR Slovenians[title/abstract] OR Ljubljana[title/abstract] OR Balkan[title/abstract] OR Balkans[title/abstract] OR Balkanic[title/abstract] OR Croatia[title/abstract] OR Croatian[title/abstract] OR Croatians[title/abstract] OR Dalmatia[title/abstract] OR Dalmatian[title/abstract] OR Dalmatians[title/abstract] OR Istria[title/abstract] OR Istrian[title/abstract] OR Istrians[title/abstract] OR Zagreb[title/abstract] OR Serbia[title/abstract] OR Serbian[title/abstract] OR Serbians[title/abstract] OR Belgrade[title/abstract] OR Belgrad[title/abstract] OR Bosnia[title/abstract] OR Bosnian[title/abstract] OR Bosnians[title/abstract] OR Herzegovina[title/abstract] OR Herzegovinian[title/abstract] OR Herzegovinians[title/abstract] OR Herzegowina[title/abstract] OR Herzegowinian[title/abstract] OR Herzegowinians[title/abstract] OR Hercegovina[title/abstract] OR Hercegovinian[title/abstract] OR Hercegovinians[title/abstract] OR Sarajevo[title/abstract] OR Montenegro[title/abstract] OR Montenegrin[title/abstract] OR Montenegrins[title/abstract] OR Montenegrian[title/abstract] OR Montenegrians[title/abstract] OR Montenegrine[title/abstract] OR Podgorica[title/abstract] OR Kosovo[title/abstract] OR Kosovan[title/abstract] OR Kosovans[title/abstract] OR Kosovar[title/abstract] OR Kosovars[title/abstract] OR Pristina[title/abstract] OR Macedonia[title/abstract] OR Macedonian[title/abstract] OR Macedonians[title/abstract] OR Skopje[title/abstract] OR Albania[title/abstract] OR Albanian[title/abstract] OR Albanians[title/abstract] OR Tirana[title/abstract] OR Greece[title/abstract] OR Greek[title/abstract] OR Greeks[title/abstract] OR Hellenic[title/abstract] OR Athens[title/abstract] OR Crete[title/abstract] OR Cretan[title/abstract] OR Cretans[title/abstract] OR Cyclades[title/abstract] OR Cycladic[title/abstract] OR Dodecanese[title/abstract] OR Dodecanesian[title/abstract] OR Peloponnese[title/abstract] OR Peloponnesian[title/abstract] OR Peloponnesians[title/abstract] OR Cyprus[title/abstract] OR Cypriot[title/abstract] OR Cypriots[title/abstract] OR Cypriotic[title/abstract] OR Cypriote[title/abstract] OR Nicosia[title/abstract] OR Malta[title/abstract] OR Maltese[title/abstract] OR Valetta[title/abstract] OR Valletta[title/abstract] OR Gozo[title/abstract] OR Gozitan[title/abstract] OR Gozitans[title/abstract] OR Portugal[title/abstract] OR Portuguese[title/abstract] OR Portugueses[title/abstract] OR Lisbon[title/abstract] OR Madeira[title/abstract] OR Madeiran[title/abstract] OR Madeirans[title/abstract] OR Azores[title/abstract] OR Azorean[title/abstract] OR Azoreans[title/abstract] OR Macaronesia[title/abstract] OR Macaronesian[title/abstract] OR Macaronesians[title/abstract] OR Alentejo[title/abstract] OR Algarve[title/abstract] OR Spain[title/abstract] OR Spanish[title/abstract] OR Iberian[title/abstract] OR Iberians[title/abstract] OR Madrid[title/abstract] OR Balearic[title/abstract] OR Majorca[title/abstract] OR Majorcan[title/abstract] OR Majorcans[title/abstract] OR Mallorca[title/abstract] OR Mallorcan[title/abstract] OR Mallorcans[title/abstract] OR Menorca[title/abstract] OR Menorcan[title/abstract] OR Menorcans[title/abstract] OR Canary[title/abstract] OR Canarian[title/abstract] OR Canarians[title/abstract] OR Andalusia[title/abstract] OR Andalusian[title/abstract] OR Andalusians[title/abstract] OR Murcia[title/abstract] OR Murcian[title/abstract] OR Murcians[title/abstract] OR Valencia[title/abstract] OR Valencian[title/abstract] OR Valencians[title/abstract] OR Castilla[title/abstract] OR Castile[title/abstract] OR Castilian[title/abstract] OR Castilians[title/abstract] OR Castillian[title/abstract] OR Castillians[title/abstract] OR Leon[title/abstract] OR Leonese[title/abstract] OR Leoneses[title/abstract] OR Extremadura[title/abstract] OR Extremaduran[title/abstract] OR Extremadurans[title/abstract] OR Catalonia[title/abstract] OR Catalan[title/abstract] OR Catalans[title/abstract] OR Aragon[title/abstract] OR Aragonese[title/abstract] OR Navarre[title/abstract] OR Navarran[title/abstract] OR Navarrans[title/abstract] OR Basque[title/abstract] OR Basques[title/abstract] OR Cantabria[title/abstract] OR Cantabric[title/abstract] OR Cantabrian[title/abstract] OR Cantabrians[title/abstract] OR Asturias[title/abstract] OR Asturian[title/abstract] OR Asturians[title/abstract] OR Galicia[title/abstract] OR Galician[title/abstract] OR Galicians[title/abstract] OR Pyrenees[title/abstract] OR Pyrenean[title/abstract] OR Gibraltar[title/abstract] OR Gibraltarian[title/abstract] OR Gibraltarians[title/abstract] OR Italy[title/abstract] OR Italian[title/abstract] OR Italians[title/abstract] OR Rome[title/abstract] OR Apennine[title/abstract] OR Padane[title/abstract] OR Sicily[title/abstract] OR Sicilian[title/abstract] OR Sicilians[title/abstract] OR Sardinia[title/abstract] OR Sardinian[title/abstract] OR Sardinians[title/abstract] OR Aosta[title/abstract] OR Trentino[title/abstract] OR Piedmont[title/abstract] OR Piedmontese[title/abstract] OR Liguria[title/abstract] OR Ligurian[title/abstract] OR Ligurians[title/abstract] OR Lombardy[title/abstract] OR Lombard[title/abstract] OR Lombards[title/abstract] OR Lombardic[title/abstract] OR Veneto[title/abstract] OR Venetian[title/abstract] OR Venetians[title/abstract] OR Friuli[title/abstract] OR Emilia-Romagna[title/abstract] OR Emilian[title/abstract] OR Tuscany[title/abstract] OR Tuscan[title/abstract] OR Tuscans[title/abstract] OR Umbria[title/abstract] OR Umbrian[title/abstract] OR Umbrians[title/abstract] OR Marche[title/abstract] OR Latium[title/abstract] OR Abruzzo[title/abstract] OR Molise[title/abstract] OR Campania[title/abstract] OR Campanian[title/abstract] OR Campanians[title/abstract] OR Apulia[title/abstract] OR Apulian[title/abstract] OR Apulians[title/abstract] OR Basilicata[title/abstract] OR Calabria[title/abstract] OR Calabrian[title/abstract] OR Calabrians[title/abstract] OR France[title/abstract] OR French[title/abstract] OR Paris[title/abstract] OR Corsica[title/abstract] OR Corsican[title/abstract] OR Corsicans[title/abstract] OR Alsace[title/abstract] OR Alsatian[title/abstract] OR Alsatians[title/abstract] OR Aquitaine[title/abstract] OR Aquitain[title/abstract] OR Aquitan[title/abstract] OR Aquitans[title/abstract] OR Occitan[title/abstract] OR Occitans[title/abstract] OR Auvergne[title/abstract] OR Auvergnese[title/abstract] OR Brittany[title/abstract] OR Bretagne[title/abstract] OR Breton[title/abstract] OR Bretons[title/abstract] OR Burgundy[title/abstract] OR Burgundian[title/abstract] OR Burgundians[title/abstract] OR Loire[title/abstract] OR Ardenne[title/abstract] OR Normandy[title/abstract] OR Norman[title/abstract] OR Normans[title/abstract] OR Franche-Comté[title/abstract] OR Languedoc-Roussillon[title/abstract] OR Limousin[title/abstract] OR Azur[title/abstract] OR Provence[title/abstract] OR Provencal[title/abstract] OR Lorraine[title/abstract] OR Lorrainian[title/abstract] OR Lorrainians[title/abstract] OR Picardy[title/abstract] OR Picard[title/abstract] OR Picards[title/abstract] OR Calais[title/abstract] OR Poitou-Charentes[title/abstract] OR Rhône-Alpes[title/abstract] OR Savoy[title/abstract] OR Savoyard[title/abstract] OR Savoyards[title/abstract] OR Austria[title/abstract] OR Austrian[title/abstract] OR Austrians[title/abstract] OR Vienna[title/abstract] OR Burgenland[title/abstract] OR Carinthia[title/abstract] OR Carinthian[title/abstract] OR Carinthians[title/abstract] OR Salzburger[title/abstract] OR Styria[title/abstract] OR Styrian[title/abstract] OR Styrians[title/abstract] OR Tyrol[title/abstract] OR Tyrolean[title/abstract] OR Tyroleans[title/abstract] OR Tyrolese[title/abstract] OR Vorarlberg[title/abstract] OR Germany[title/abstract] OR German[title/abstract] OR Berlin[title/abstract] OR Baden-Württemberg[title/abstract] OR Bavaria[title/abstract] OR Bavarian[title/abstract] OR Bavarians[title/abstract] OR Brandenburg[title/abstract] OR Bremen[title/abstract] OR Hamburg[title/abstract] OR Hesse[title/abstract] OR Hessian[title/abstract] OR Hessians[title/abstract] OR Holstein[title/abstract] OR Holsteinian[title/abstract] OR Holsteinians[title/abstract] OR Saxony[title/abstract] OR Saxon[title/abstract] OR Saxons[title/abstract] OR Mecklenburg[title/abstract] OR Westphalia[title/abstract] OR Westphalian[title/abstract] OR Westphalians[title/abstract] OR Palatinate[title/abstract] OR Palatine[title/abstract] OR Palatines[title/abstract] OR Saarland[title/abstract] OR Thuringia[title/abstract] OR Thuringian[title/abstract] OR Thuringians[title/abstract] OR Franconia[title/abstract] OR Franconian[title/abstract] OR Franconians[title/abstract] OR Lusatia[title/abstract] OR Lusatian[title/abstract] OR Lusatians[title/abstract] OR Rhineland[title/abstract] OR Rhenish[title/abstract] OR Rhinelanders[title/abstract] OR Switzerland[title/abstract] OR Swiss[title/abstract] OR Helvetian[title/abstract] OR Helvetians[title/abstract] OR Bern[title/abstract] OR Berne[title/abstract] OR Aargau[title/abstract] OR "Appenzell Ausserrhoden"[title/abstract] OR Basel[title/abstract] OR Geneva[title/abstract] OR Glarus[title/abstract] OR Grisons[title/abstract] OR Jura[title/abstract] OR Lucerne[title/abstract] OR Neuchatel[title/abstract] OR Nidwalden[title/abstract] OR Obwalden[title/abstract] OR Schaffhausen[title/abstract] OR Schwyz[title/abstract] OR Solothurn[title/abstract] OR Gallen[title/abstract] OR Thurgau[title/abstract] OR Ticino[title/abstract] OR Tessin[title/abstract] OR Uri[title/abstract] OR Valais[title/abstract] OR Vaud[title/abstract] OR Zug[title/abstract] OR Zurich[title/abstract] OR Iceland[title/abstract] OR Icelander[title/abstract] OR Icelanders[title/abstract] OR Icelandic[title/abstract] OR Reykjavik[title/abstract] OR Ireland[title/abstract] OR Irish[title/abstract] OR Eire[title/abstract] OR Dublin[title/abstract] OR Kerry[title/abstract] OR Donegal[title/abstract] OR Connemara[title/abstract] OR Shannon[title/abstract] OR Aran[title/abstract] OR Celtic[title/abstract] OR Gaelic[title/abstract] OR "Great-Britain"[title/abstract] OR "United Kingdom"[title/abstract] OR British[title/abstract] OR London[title/abstract] OR England[title/abstract] OR English[title/abstract] OR Cornwall[title/abstract] OR Cornish[title/abstract] OR Scotland[title/abstract] OR Scottish[title/abstract] OR Wales[title/abstract] OR Welsh[title/abstract] OR Ulster[title/abstract] OR "Channel Islands"[title/abstract] OR Guernsey[title/abstract] OR "Isle of Man"[title/abstract] OR Orkney[title/abstract] OR Orcadian[title/abstract] OR Orcadians[title/abstract] OR Hebrides[title/abstract] OR Hebridean[title/abstract] OR Hebrideans[title/abstract] OR Shetland[title/abstract] OR Shetlanders[title/abstract] OR Cumbria[title/abstract] OR Cumbrian[title/abstract] OR Cumbrians[title/abstract] OR Fife[title/abstract] OR Fifers[title/abstract] OR Lancashire[title/abstract] OR Lancastrian[title/abstract] OR Lancastrians[title/abstract] OR Northumberland[title/abstract] OR Northumbria[title/abstract] OR Northumbrian[title/abstract] OR Northumbrians[title/abstract] OR Scandinavia[title/abstract] OR Scandinavian[title/abstract] OR Scandinavians[title/abstract] OR Denmark[title/abstract] OR Danish[title/abstract] OR Copenhagen[title/abstract] OR Jutland[title/abstract] OR Jutlanders[title/abstract] OR "Region-Zealand"[title/abstract] OR Faeroe[title/abstract] OR Finland[title/abstract] OR Finnish[title/abstract] OR Finnic[title/abstract] OR Helsinki[title/abstract] OR Lapland[title/abstract] OR Lapp[title/abstract] OR Lappish[title/abstract] OR Saami[title/abstract] OR Saamis[title/abstract] OR Ostrobothnia[title/abstract] OR Kainuu[title/abstract] OR Savo[title/abstract] OR Pirkanmaa[title/abstract] OR Satakunta[title/abstract] OR Paijat-Hame[title/abstract] OR Kanta-Hame[title/abstract] OR Kymenlaakso[title/abstract] OR Uusimaa[title/abstract] OR Aland[title/abstract] OR Norway[title/abstract] OR Norwegian[title/abstract] OR Norwegians[title/abstract] OR Oslo[title/abstract] OR Svalbard[title/abstract] OR "Jan Mayen"[title/abstract] OR Troms[title/abstract] OR Nordland[title/abstract] OR Trondelag[title/abstract] OR Romsdal[title/abstract] OR Rogaland[title/abstract] OR Agder[title/abstract] OR Vestfold[title/abstract] OR Viken[title/abstract] OR Innlandet[title/abstract] OR Lofoten[title/abstract] OR Sweden[title/abstract] OR Swedish[title/abstract] OR Stockholm[title/abstract] OR Gotland[title/abstract] OR Gotlanders[title/abstract] OR Oland[title/abstract] OR Scania[title/abstract] OR Scanian[title/abstract] OR Scanians[title/abstract] OR Skane[title/abstract] OR Norrland[title/abstract] OR Svealand[title/abstract] OR Götaland[title/abstract] OR Blekinge[title/abstract] OR Dalarna[title/abstract] OR Gavleborg[title/abstract] OR Uppsala[title/abstract] OR Halland[title/abstract] OR Jamtland[title/abstract] OR Jonkoping[title/abstract] OR Kalmar[title/abstract] OR Kronoberg[title/abstract] OR Norrbotten[title/abstract] OR Sodermanland[title/abstract] OR Varmland[title/abstract] OR Vasterbotten[title/abstract] OR Vasternorrland[title/abstract] OR Vastmanland[title/abstract] OR Vastra[title/abstract] OR Orebro[title/abstract] OR Ostergotland[title/abstract] OR Netherland*[title/abstract] OR Holland[title/abstract] OR Amsterdam[title/abstract] OR Zeeland[title/abstract] OR Zeelanders[title/abstract] OR Friesland[title/abstract] OR Friesian[title/abstract] OR Friesians[title/abstract] OR Frisian[title/abstract] OR Frisians[title/abstract] OR Drenthe[title/abstract] OR Gelderland[title/abstract] OR Flevoland[title/abstract] OR Limburg[title/abstract] OR Noord-Brabant[title/abstract] OR Overijssel[title/abstract] OR Utrecht[title/abstract] OR Belgium[title/abstract] OR Belgian[title/abstract] OR Belgians[title/abstract] OR Brussels[title/abstract] OR Flanders[title/abstract] OR Flemish[title/abstract] OR Flemings[title/abstract] OR Wallonia[title/abstract] OR Walloon[title/abstract] OR Walloons[title/abstract] OR Luxembourg*[title/abstract])

AND

(epidemiology[subheading] OR "Epidemiology"[Mesh] OR epidemiol*[title/abstract] OR statistic*[title/abstract] OR "Incidence"[Mesh] OR incidence*[title/abstract] OR rate[title/abstract] OR rates[title/abstract] OR distribution*[title/abstract] OR cases[title/abstract] OR "Prevalence"[Mesh] OR prevalen*[title/abstract] OR "Gene Frequency"[Mesh] OR frequenc*[title/abstract] OR occurrenc*[title/abstract] OR "Observational Study"[Publication Type] OR "Epidemiologic Studies"[Mesh] OR observational[title/abstract] OR case-control*[title/abstract] OR retrospective*[title/abstract] OR cohort*[title/abstract] OR longitudinal*[title/abstract] OR prospective*[title/abstract] OR cross-section*[title/abstract] OR transversal*[title/abstract] OR population-based[title/abstract] OR series[title/abstract] OR "Regression Analysis"[Mesh] OR regression[title/abstract] OR "Matched-Pair Analysis"[Mesh] OR matching[title/abstract] OR matched[title/abstract])

# **Supplementary material 1B. Search string used in Embase for the literature research.**

('dominant cerebellar ataxia*':ti,ab,kw OR ADCA:ti,ab,kw OR ADCAs:ti,ab,kw OR 'spinocerebellar ataxia 1'/exp OR 'spinocerebellar ataxia 1':ti,ab,kw OR 'spino-cerebellar ataxia 1':ti,ab,kw OR SCA1:ti,ab,kw OR 'spinocerebellar ataxia 2'/exp OR 'spinocerebellar ataxia 2':ti,ab,kw OR 'spino-cerebellar ataxia 2':ti,ab,kw OR SCA2:ti,ab,kw OR 'spinocerebellar ataxia 3'/exp OR 'Machado Joseph disease'/exp OR 'spinocerebellar ataxia 3':ti,ab,kw OR 'spino-cerebellar ataxia 3':ti,ab,kw OR SCA3:ti,ab,kw OR 'Machado-Joseph':ti,ab,kw OR 'Joseph disease':ti,ab,kw OR 'Machado disease':ti,ab,kw OR 'Machado syndrome':ti,ab,kw OR 'Azorean disease':ti,ab,kw OR 'Azorean neurologic disease':ti,ab,kw OR 'Azorean ataxia':ti,ab,kw OR 'nigrospinodentatal degeneration*':ti,ab,kw OR 'nigro-spino-dentatal degeneration*':ti,ab,kw OR 'spinocerebellar ataxia 6'/exp OR 'spinocerebellar ataxia 6':ti,ab,kw OR 'spino-cerebellar ataxia 6':ti,ab,kw OR SCA6:ti,ab,kw OR 'spinocerebellar ataxia 7'/exp OR 'spinocerebellar ataxia 7':ti,ab,kw OR 'spino-cerebellar ataxia 7':ti,ab,kw OR SCA7:ti,ab,kw OR ('pigmentary retinopath*':ti,ab,kw OR 'pigmentary maculopath*':ti,ab,kw OR 'retinal degenerat*':ti,ab,kw AND ataxi*:ti,ab,kw) OR 'spinocerebellar ataxia 8':ti,ab,kw OR 'spino-cerebellar ataxia 8':ti,ab,kw OR SCA8:ti,ab,kw OR 'spinocerebellar ataxia 17':ti,ab,kw OR 'spino-cerebellar ataxia 17':ti,ab,kw OR SCA17:ti,ab,kw OR 'Huntington Disease-Like 4':ti,ab,kw OR HDL4:ti,ab,kw OR (('spinocerebellar degeneration'/exp OR 'spinocerebellar ataxia*':ti,ab,kw OR 'spinocerebellar degeneration*':ti,ab,kw OR 'spinocerebellar disease*':ti,ab,kw OR 'spino-cerebellar ataxia*':ti,ab,kw OR 'spino-cerebellar degeneration*':ti,ab,kw OR 'spino-cerebellar disease*':ti,ab,kw) AND ('type 1':ti,ab,kw OR 'type 2':ti,ab,kw OR 'type 3':ti,ab,kw OR 'type 6':ti,ab,kw OR 'type 7':ti,ab,kw OR 'type 8':ti,ab,kw OR 'type 9':ti,ab,kw OR 'type I':ti,ab,kw OR 'type II':ti,ab,kw OR 'type III':ti,ab,kw OR 'type VI':ti,ab,kw OR 'type VII':ti,ab,kw OR 'type VIII':ti,ab,kw OR 'type IX':ti,ab,kw)) OR 'sca6 gene'/exp OR 'sca17 gene'/exp OR (('ataxin 1'/exp OR 'ataxin-1':ti,ab,kw OR 'atxn-1':ti,ab,kw OR atxn1:ti,ab,kw OR 'ataxin 2'/exp OR 'ataxin-2':ti,ab,kw OR 'atxn-2':ti,ab,kw OR atxn2:ti,ab,kw OR 'atx-2':ti,ab,kw OR 'ataxin 3'/exp OR 'ataxin-3':ti,ab,kw OR 'atxn-3':ti,ab,kw OR atxn3:ti,ab,kw OR CACNA1A:ti,ab,kw OR 'ataxin 7'/exp OR 'ataxin-7':ti,ab,kw OR 'atxn-7':ti,ab,kw OR atxn7:ti,ab,kw OR 'ataxin-8':ti,ab,kw OR 'atxn-8':ti,ab,kw OR atxn8:ti,ab,kw OR 'TATA binding protein related factor'/exp OR 'TATA Box':ti,ab,kw OR 'TBP gene':ti,ab,kw) AND ('cerebellar ataxia'/exp OR 'spinocerebellar degeneration'/exp OR ataxia*:ti,ab,kw OR cerebell*:ti,ab,kw OR spinocerebell*:ti,ab,kw)))

AND

('global disease burden'/exp OR 'global health'/exp OR global:ti,ab,kw OR worldwide:ti,ab,kw OR world-wide:ti,ab,kw OR 'Europe'/exp OR 'European'/exp OR Europe:ti,ab,kw OR European:ti,ab,kw OR Europeans:ti,ab,kw OR 'Alpine region*':ti,ab,kw OR 'Alpine area*':ti,ab,kw OR 'Alpine countr*':ti,ab,kw OR 'Mediterranean region*':ti,ab,kw OR 'Mediterranean area*':ti,ab,kw OR 'Mediterranean countr*':ti,ab,kw OR Baltic:ti,ab,kw OR Baltics:ti,ab,kw OR Balts:ti,ab,kw OR Estonia:ti,ab,kw OR Estonian:ti,ab,kw OR Estonians:ti,ab,kw OR Tallin:ti,ab,kw OR Latvia:ti,ab,kw OR Latvian:ti,ab,kw OR Latvians:ti,ab,kw OR Letts:ti,ab,kw OR Riga:ti,ab,kw OR Courland:ti,ab,kw OR Curonian:ti,ab,kw OR Curonians:ti,ab,kw OR Lithuania:ti,ab,kw OR Lithuanian:ti,ab,kw OR Lithuanians:ti,ab,kw OR Vilnius:ti,ab,kw OR Russia:ti,ab,kw OR Russian:ti,ab,kw OR Russians:ti,ab,kw OR Moskow:ti,ab,kw OR Bashkiria:ti,ab,kw OR Bashkirian:ti,ab,kw OR Bashkirians:ti,ab,kw OR Bashkortostan:ti,ab,kw OR Dagestan:ti,ab,kw OR Dagestani:ti,ab,kw OR Dagestanian:ti,ab,kw OR Dagestanians:ti,ab,kw OR Daghestan:ti,ab,kw OR Daghestani:ti,ab,kw OR Daghestanian:ti,ab,kw OR Daghestanians:ti,ab,kw OR Tatar:ti,ab,kw OR Tatars:ti,ab,kw OR Siberia:ti,ab,kw OR Siberian:ti,ab,kw OR Siberians:ti,ab,kw OR Karelia:ti,ab,kw OR Karelian:ti,ab,kw OR Karelians:ti,ab,kw OR Carelia:ti,ab,kw OR Carelian:ti,ab,kw OR Carelians:ti,ab,kw OR Crimea:ti,ab,kw OR Crimean:ti,ab,kw OR Crimeans:ti,ab,kw OR Armenia:ti,ab,kw OR Armenian:ti,ab,kw OR Armenians:ti,ab,kw OR Transcaucasia:ti,ab,kw OR Transcaucasian:ti,ab,kw OR Transcaucasians:ti,ab,kw OR Azerbaijan:ti,ab,kw OR Azerbaijani:ti,ab,kw OR Azerbaijanis:ti,ab,kw OR Nagorno-Karabakh:ti,ab,kw OR Artsakh*:ti,ab,kw OR 'Georgian Republic':ti,ab,kw OR Abkhasia:ti,ab,kw OR Abkhasian:ti,ab,kw OR Abkhasians:ti,ab,kw OR Ossetia:ti,ab,kw OR Ossetian:ti,ab,kw OR Ossetians:ti,ab,kw OR Belarus:ti,ab,kw OR Belarussian:ti,ab,kw OR Belarussians:ti,ab,kw OR Byelorussia:ti,ab,kw OR Byelorussian:ti,ab,kw OR Byelorussians:ti,ab,kw OR Belorussia:ti,ab,kw OR Belorussian:ti,ab,kw OR Belorussians:ti,ab,kw OR Byelarus:ti,ab,kw OR Byelarussian:ti,ab,kw OR Byelarussians:ti,ab,kw OR Belorussia:ti,ab,kw OR Belorussian:ti,ab,kw OR Belorussians:ti,ab,kw OR Minsk:ti,ab,kw OR Ukraine:ti,ab,kw OR Ukrainian:ti,ab,kw OR Ukrainians:ti,ab,kw OR Kiev:ti,ab,kw OR Bessarabia:ti,ab,kw OR Bessarabian:ti,ab,kw OR Bessarabians:ti,ab,kw OR Bukovina:ti,ab,kw OR Bukovinian:ti,ab,kw OR Bukovinians:ti,ab,kw OR Crimea:ti,ab,kw OR Crimean:ti,ab,kw OR Crimeans:ti,ab,kw OR Moldova:ti,ab,kw OR Moldovan:ti,ab,kw OR Moldovans:ti,ab,kw OR Moldavia:ti,ab,kw OR Moldavian:ti,ab,kw OR Moldavians:ti,ab,kw OR Chisinau:ti,ab,kw OR Poland:ti,ab,kw OR Polish:ti,ab,kw OR Polska:ti,ab,kw OR Warsaw:ti,ab,kw OR Warszaw:ti,ab,kw OR Lemko:ti,ab,kw OR Lemkos:ti,ab,kw OR Lemkian:ti,ab,kw OR Lemkians:ti,ab,kw OR Lemk:ti,ab,kw OR Lemks:ti,ab,kw OR Pomerania:ti,ab,kw OR Pomeranian:ti,ab,kw OR Pomeranians:ti,ab,kw OR Silesia:ti,ab,kw OR Silesian:ti,ab,kw OR Silesians:ti,ab,kw OR Moravia:ti,ab,kw OR Moravian:ti,ab,kw OR Moravians:ti,ab,kw OR Bulgaria:ti,ab,kw OR Bulgarian:ti,ab,kw OR Bulgarians:ti,ab,kw OR Sofia:ti,ab,kw OR Thrace:ti,ab,kw OR Thracian:ti,ab,kw OR Thracians:ti,ab,kw OR Romania:ti,ab,kw OR Romanian:ti,ab,kw OR Romanians:ti,ab,kw OR Rumania:ti,ab,kw OR Rumanian:ti,ab,kw OR Rumanians:ti,ab,kw OR Bucharest:ti,ab,kw OR Bucuresti:ti,ab,kw OR Transylvania:ti,ab,kw OR Transylvanian:ti,ab,kw OR Transylvanians:ti,ab,kw OR Transilvania:ti,ab,kw OR Transilvanian:ti,ab,kw OR Transilvanians:ti,ab,kw OR Wallachia:ti,ab,kw OR Wallachian:ti,ab,kw OR Wallachians:ti,ab,kw OR Hungary:ti,ab,kw OR Hungarian:ti,ab,kw OR Hungarians:ti,ab,kw OR Magyar:ti,ab,kw OR Magyars:ti,ab,kw OR Budapest:ti,ab,kw OR Swabia:ti,ab,kw OR Swabian:ti,ab,kw OR Swabians:ti,ab,kw OR Slovakia:ti,ab,kw OR Slovak:ti,ab,kw OR Slovaks:ti,ab,kw OR Slovakian:ti,ab,kw OR Slovakians:ti,ab,kw OR Bratislava:ti,ab,kw OR Czech:ti,ab,kw OR Czechia:ti,ab,kw OR Czechs:ti,ab,kw OR Bohemia:ti,ab,kw OR Bohemian:ti,ab,kw OR Bohemians:ti,ab,kw OR Prague:ti,ab,kw OR Slovenia:ti,ab,kw OR Slovenian:ti,ab,kw OR Slovenians:ti,ab,kw OR Ljubljana:ti,ab,kw OR Balkan:ti,ab,kw OR Balkans:ti,ab,kw OR Balkanic:ti,ab,kw OR Croatia:ti,ab,kw OR Croatian:ti,ab,kw OR Croatians:ti,ab,kw OR Dalmatia:ti,ab,kw OR Dalmatian:ti,ab,kw OR Dalmatians:ti,ab,kw OR Istria:ti,ab,kw OR Istrian:ti,ab,kw OR Istrians:ti,ab,kw OR Zagreb:ti,ab,kw OR Serbia:ti,ab,kw OR Serbian:ti,ab,kw OR Serbians:ti,ab,kw OR Belgrade:ti,ab,kw OR Belgrad:ti,ab,kw OR Bosnia:ti,ab,kw OR Bosnian:ti,ab,kw OR Bosnians:ti,ab,kw OR Herzegovina:ti,ab,kw OR Herzegovinian:ti,ab,kw OR Herzegovinians:ti,ab,kw OR Herzegowina:ti,ab,kw OR Herzegowinian:ti,ab,kw OR Herzegowinians:ti,ab,kw OR Hercegovina:ti,ab,kw OR Hercegovinian:ti,ab,kw OR Hercegovinians:ti,ab,kw OR Sarajevo:ti,ab,kw OR Montenegro:ti,ab,kw OR Montenegrin:ti,ab,kw OR Montenegrins:ti,ab,kw OR Montenegrian:ti,ab,kw OR Montenegrians:ti,ab,kw OR Montenegrine:ti,ab,kw OR Podgorica:ti,ab,kw OR Kosovo:ti,ab,kw OR Kosovan:ti,ab,kw OR Kosovans:ti,ab,kw OR Kosovar:ti,ab,kw OR Kosovars:ti,ab,kw OR Pristina:ti,ab,kw OR Macedonia:ti,ab,kw OR Macedonian:ti,ab,kw OR Macedonians:ti,ab,kw OR Skopje:ti,ab,kw OR Albania:ti,ab,kw OR Albanian:ti,ab,kw OR Albanians:ti,ab,kw OR Tirana:ti,ab,kw OR Greece:ti,ab,kw OR Greek:ti,ab,kw OR Greeks:ti,ab,kw OR Hellenic:ti,ab,kw OR Athens:ti,ab,kw OR Crete:ti,ab,kw OR Cretan:ti,ab,kw OR Cretans:ti,ab,kw OR Cyclades:ti,ab,kw OR Cycladic:ti,ab,kw OR Dodecanese:ti,ab,kw OR Dodecanesian:ti,ab,kw OR Peloponnese:ti,ab,kw OR Peloponnesian:ti,ab,kw OR Peloponnesians:ti,ab,kw OR Cyprus:ti,ab,kw OR Cypriot:ti,ab,kw OR Cypriots:ti,ab,kw OR Cypriotic:ti,ab,kw OR Cypriote:ti,ab,kw OR Nicosia:ti,ab,kw OR Malta:ti,ab,kw OR Maltese:ti,ab,kw OR Valetta:ti,ab,kw OR Valletta:ti,ab,kw OR Gozo:ti,ab,kw OR Gozitan:ti,ab,kw OR Gozitans:ti,ab,kw OR Portugal:ti,ab,kw OR Portuguese:ti,ab,kw OR Portugueses:ti,ab,kw OR Lisbon:ti,ab,kw OR Madeira:ti,ab,kw OR Madeiran:ti,ab,kw OR Madeirans:ti,ab,kw OR Azores:ti,ab,kw OR Azorean:ti,ab,kw OR Azoreans:ti,ab,kw OR Macaronesia:ti,ab,kw OR Macaronesian:ti,ab,kw OR Macaronesians:ti,ab,kw OR Alentejo:ti,ab,kw OR Algarve:ti,ab,kw OR Spain:ti,ab,kw OR Spanish:ti,ab,kw OR Iberian:ti,ab,kw OR Iberians:ti,ab,kw OR Madrid:ti,ab,kw OR Balearic:ti,ab,kw OR Majorca:ti,ab,kw OR Majorcan:ti,ab,kw OR Majorcans:ti,ab,kw OR Mallorca:ti,ab,kw OR Mallorcan:ti,ab,kw OR Mallorcans:ti,ab,kw OR Menorca:ti,ab,kw OR Menorcan:ti,ab,kw OR Menorcans:ti,ab,kw OR Canary:ti,ab,kw OR Canarian:ti,ab,kw OR Canarians:ti,ab,kw OR Andalusia:ti,ab,kw OR Andalusian:ti,ab,kw OR Andalusians:ti,ab,kw OR Murcia:ti,ab,kw OR Murcian:ti,ab,kw OR Murcians:ti,ab,kw OR Valencia:ti,ab,kw OR Valencian:ti,ab,kw OR Valencians:ti,ab,kw OR Castilla:ti,ab,kw OR Castile:ti,ab,kw OR Castilian:ti,ab,kw OR Castilians:ti,ab,kw OR Castillian:ti,ab,kw OR Castillians:ti,ab,kw OR Leon:ti,ab,kw OR Leonese:ti,ab,kw OR Leoneses:ti,ab,kw OR Extremadura:ti,ab,kw OR Extremaduran:ti,ab,kw OR Extremadurans:ti,ab,kw OR Catalonia:ti,ab,kw OR Catalan:ti,ab,kw OR Catalans:ti,ab,kw OR Aragon:ti,ab,kw OR Aragonese:ti,ab,kw OR Navarre:ti,ab,kw OR Navarran:ti,ab,kw OR Navarrans:ti,ab,kw OR Basque:ti,ab,kw OR Basques:ti,ab,kw OR Cantabria:ti,ab,kw OR Cantabric:ti,ab,kw OR Cantabrian:ti,ab,kw OR Cantabrians:ti,ab,kw OR Asturias:ti,ab,kw OR Asturian:ti,ab,kw OR Asturians:ti,ab,kw OR Galicia:ti,ab,kw OR Galician:ti,ab,kw OR Galicians:ti,ab,kw OR Pyrenees:ti,ab,kw OR Pyrenean:ti,ab,kw OR Gibraltar:ti,ab,kw OR Gibraltarian:ti,ab,kw OR Gibraltarians:ti,ab,kw OR Italy:ti,ab,kw OR Italian:ti,ab,kw OR Italians:ti,ab,kw OR Rome:ti,ab,kw OR Apennine:ti,ab,kw OR Padane:ti,ab,kw OR Sicily:ti,ab,kw OR Sicilian:ti,ab,kw OR Sicilians:ti,ab,kw OR Sardinia:ti,ab,kw OR Sardinian:ti,ab,kw OR Sardinians:ti,ab,kw OR Aosta:ti,ab,kw OR Trentino:ti,ab,kw OR Piedmont:ti,ab,kw OR Piedmontese:ti,ab,kw OR Liguria:ti,ab,kw OR Ligurian:ti,ab,kw OR Ligurians:ti,ab,kw OR Lombardy:ti,ab,kw OR Lombard:ti,ab,kw OR Lombards:ti,ab,kw OR Lombardic:ti,ab,kw OR Veneto:ti,ab,kw OR Venetian:ti,ab,kw OR Venetians:ti,ab,kw OR Friuli:ti,ab,kw OR Emilia-Romagna:ti,ab,kw OR Emilian:ti,ab,kw OR Tuscany:ti,ab,kw OR Tuscan:ti,ab,kw OR Tuscans:ti,ab,kw OR Umbria:ti,ab,kw OR Umbrian:ti,ab,kw OR Umbrians:ti,ab,kw OR Marche:ti,ab,kw OR Latium:ti,ab,kw OR Abruzzo:ti,ab,kw OR Molise:ti,ab,kw OR Campania:ti,ab,kw OR Campanian:ti,ab,kw OR Campanians:ti,ab,kw OR Apulia:ti,ab,kw OR Apulian:ti,ab,kw OR Apulians:ti,ab,kw OR Basilicata:ti,ab,kw OR Calabria:ti,ab,kw OR Calabrian:ti,ab,kw OR Calabrians:ti,ab,kw OR France:ti,ab,kw OR French:ti,ab,kw OR Paris:ti,ab,kw OR Corsica:ti,ab,kw OR Corsican:ti,ab,kw OR Corsicans:ti,ab,kw OR Alsace:ti,ab,kw OR Alsatian:ti,ab,kw OR Alsatians:ti,ab,kw OR Aquitaine:ti,ab,kw OR Aquitain:ti,ab,kw OR Aquitan:ti,ab,kw OR Aquitans:ti,ab,kw OR Occitan:ti,ab,kw OR Occitans:ti,ab,kw OR Auvergne:ti,ab,kw OR Auvergnese:ti,ab,kw OR Brittany:ti,ab,kw OR Bretagne:ti,ab,kw OR Breton:ti,ab,kw OR Bretons:ti,ab,kw OR Burgundy:ti,ab,kw OR Burgundian:ti,ab,kw OR Burgundians:ti,ab,kw OR Loire:ti,ab,kw OR Ardenne:ti,ab,kw OR Normandy:ti,ab,kw OR Norman:ti,ab,kw OR Normans:ti,ab,kw OR Franche-Comté:ti,ab,kw OR Languedoc-Roussillon:ti,ab,kw OR Limousin:ti,ab,kw OR Azur:ti,ab,kw OR Provence:ti,ab,kw OR Provencal:ti,ab,kw OR Lorraine:ti,ab,kw OR Lorrainian:ti,ab,kw OR Lorrainians:ti,ab,kw OR Picardy:ti,ab,kw OR Picard:ti,ab,kw OR Picards:ti,ab,kw OR Calais:ti,ab,kw OR Poitou-Charentes:ti,ab,kw OR Rhone-Alpes:ti,ab,kw OR Savoy:ti,ab,kw OR Savoyard:ti,ab,kw OR Savoyards:ti,ab,kw OR Austria:ti,ab,kw OR Austrian:ti,ab,kw OR Austrians:ti,ab,kw OR Vienna:ti,ab,kw OR Burgenland:ti,ab,kw OR Carinthia:ti,ab,kw OR Carinthian:ti,ab,kw OR Carinthians:ti,ab,kw OR Salzburger:ti,ab,kw OR Styria:ti,ab,kw OR Styrian:ti,ab,kw OR Styrians:ti,ab,kw OR Tyrol:ti,ab,kw OR Tyrolean:ti,ab,kw OR Tyroleans:ti,ab,kw OR Tyrolese:ti,ab,kw OR Vorarlberg:ti,ab,kw OR Germany:ti,ab,kw OR German:ti,ab,kw OR Berlin:ti,ab,kw OR Baden-Wurttemberg:ti,ab,kw OR Bavaria:ti,ab,kw OR Bavarian:ti,ab,kw OR Bavarians:ti,ab,kw OR Brandenburg:ti,ab,kw OR Bremen:ti,ab,kw OR Hamburg:ti,ab,kw OR Hesse:ti,ab,kw OR Hessian:ti,ab,kw OR Hessians:ti,ab,kw OR Holstein:ti,ab,kw OR Holsteinian:ti,ab,kw OR Holsteinians:ti,ab,kw OR Saxony:ti,ab,kw OR Saxon:ti,ab,kw OR Saxons:ti,ab,kw OR Mecklenburg:ti,ab,kw OR Westphalia:ti,ab,kw OR Westphalian:ti,ab,kw OR Westphalians:ti,ab,kw OR Palatinate:ti,ab,kw OR Palatine:ti,ab,kw OR Palatines:ti,ab,kw OR Saarland:ti,ab,kw OR Thuringia:ti,ab,kw OR Thuringian:ti,ab,kw OR Thuringians:ti,ab,kw OR Franconia:ti,ab,kw OR Franconian:ti,ab,kw OR Franconians:ti,ab,kw OR Lusatia:ti,ab,kw OR Lusatian:ti,ab,kw OR Lusatians:ti,ab,kw OR Rhineland:ti,ab,kw OR Rhenish:ti,ab,kw OR Rhinelanders:ti,ab,kw OR Switzerland:ti,ab,kw OR Swiss:ti,ab,kw OR Helvetian:ti,ab,kw OR Helvetians:ti,ab,kw OR Bern:ti,ab,kw OR Berne:ti,ab,kw OR Aargau:ti,ab,kw OR 'Appenzell Ausserrhoden':ti,ab,kw OR Basel:ti,ab,kw OR Geneva:ti,ab,kw OR Glarus:ti,ab,kw OR Grisons:ti,ab,kw OR Jura:ti,ab,kw OR Lucerne:ti,ab,kw OR Neuchatel:ti,ab,kw OR Nidwalden:ti,ab,kw OR Obwalden:ti,ab,kw OR Schaffhausen:ti,ab,kw OR Schwyz:ti,ab,kw OR Solothurn:ti,ab,kw OR Gallen:ti,ab,kw OR Thurgau:ti,ab,kw OR Ticino:ti,ab,kw OR Tessin:ti,ab,kw OR Uri:ti,ab,kw OR Valais:ti,ab,kw OR Vaud:ti,ab,kw OR Zug:ti,ab,kw OR Zurich:ti,ab,kw OR Iceland:ti,ab,kw OR Icelander:ti,ab,kw OR Icelanders:ti,ab,kw OR Icelandic:ti,ab,kw OR Reykjavik:ti,ab,kw OR Ireland:ti,ab,kw OR Irish:ti,ab,kw OR Eire:ti,ab,kw OR Dublin:ti,ab,kw OR Kerry:ti,ab,kw OR Donegal:ti,ab,kw OR Connemara:ti,ab,kw OR Shannon:ti,ab,kw OR Aran:ti,ab,kw OR Celtic:ti,ab,kw OR Gaelic:ti,ab,kw OR 'Great-Britain':ti,ab,kw OR 'United Kingdom':ti,ab,kw OR British:ti,ab,kw OR London:ti,ab,kw OR England:ti,ab,kw OR English:ti,ab,kw OR Cornwall:ti,ab,kw OR Cornish:ti,ab,kw OR Scotland:ti,ab,kw OR Scottish:ti,ab,kw OR Wales:ti,ab,kw OR Welsh:ti,ab,kw OR Ulster:ti,ab,kw OR 'Channel Islands':ti,ab,kw OR Guernsey:ti,ab,kw OR 'Isle of Man':ti,ab,kw OR Orkney:ti,ab,kw OR Orcadian:ti,ab,kw OR Orcadians:ti,ab,kw OR Hebrides:ti,ab,kw OR Hebridean:ti,ab,kw OR Hebrideans:ti,ab,kw OR Shetland:ti,ab,kw OR Shetlanders:ti,ab,kw OR Cumbria:ti,ab,kw OR Cumbrian:ti,ab,kw OR Cumbrians:ti,ab,kw OR Fife:ti,ab,kw OR Fifers:ti,ab,kw OR Lancashire:ti,ab,kw OR Lancastrian:ti,ab,kw OR Lancastrians:ti,ab,kw OR Northumberland:ti,ab,kw OR Northumbria:ti,ab,kw OR Northumbrian:ti,ab,kw OR Northumbrians:ti,ab,kw OR Scandinavia:ti,ab,kw OR Scandinavian:ti,ab,kw OR Scandinavians:ti,ab,kw OR Denmark:ti,ab,kw OR Danish:ti,ab,kw OR Copenhagen:ti,ab,kw OR Jutland:ti,ab,kw OR Jutlanders:ti,ab,kw OR 'Region-Zealand':ti,ab,kw OR Faeroe:ti,ab,kw OR Finland:ti,ab,kw OR Finnish:ti,ab,kw OR Finnic:ti,ab,kw OR Helsinki:ti,ab,kw OR Lapland:ti,ab,kw OR Lapp:ti,ab,kw OR Lappish:ti,ab,kw OR Saami:ti,ab,kw OR Saamis:ti,ab,kw OR Ostrobothnia:ti,ab,kw OR Kainuu:ti,ab,kw OR Savo:ti,ab,kw OR Pirkanmaa:ti,ab,kw OR Satakunta:ti,ab,kw OR Paijat-Hame:ti,ab,kw OR Kanta-Hame:ti,ab,kw OR Kymenlaakso:ti,ab,kw OR Uusimaa:ti,ab,kw OR Aland:ti,ab,kw OR Norway:ti,ab,kw OR Norwegian:ti,ab,kw OR Norwegians:ti,ab,kw OR Oslo:ti,ab,kw OR Svalbard:ti,ab,kw OR 'Jan Mayen':ti,ab,kw OR Troms:ti,ab,kw OR Nordland:ti,ab,kw OR Trondelag:ti,ab,kw OR Romsdal:ti,ab,kw OR Rogaland:ti,ab,kw OR Agder:ti,ab,kw OR Vestfold:ti,ab,kw OR Viken:ti,ab,kw OR Innlandet:ti,ab,kw OR Lofoten:ti,ab,kw OR Sweden:ti,ab,kw OR Swedish:ti,ab,kw OR Stockholm:ti,ab,kw OR Gotland:ti,ab,kw OR Gotlanders:ti,ab,kw OR Oland:ti,ab,kw OR Scania:ti,ab,kw OR Scanian:ti,ab,kw OR Scanians:ti,ab,kw OR Skane:ti,ab,kw OR Norrland:ti,ab,kw OR Svealand:ti,ab,kw OR Götaland:ti,ab,kw OR Blekinge:ti,ab,kw OR Dalarna:ti,ab,kw OR Gavleborg:ti,ab,kw OR Uppsala:ti,ab,kw OR Halland:ti,ab,kw OR Jamtland:ti,ab,kw OR Jonkoping:ti,ab,kw OR Kalmar:ti,ab,kw OR Kronoberg:ti,ab,kw OR Norrbotten:ti,ab,kw OR Sodermanland:ti,ab,kw OR Varmland:ti,ab,kw OR Vasterbotten:ti,ab,kw OR Vasternorrland:ti,ab,kw OR Vastmanland:ti,ab,kw OR Vastra:ti,ab,kw OR Orebro:ti,ab,kw OR Ostergotland:ti,ab,kw OR Netherland*:ti,ab,kw OR Holland:ti,ab,kw OR Amsterdam:ti,ab,kw OR Zeeland:ti,ab,kw OR Zeelanders:ti,ab,kw OR Friesland:ti,ab,kw OR Friesian:ti,ab,kw OR Friesians:ti,ab,kw OR Frisian:ti,ab,kw OR Frisians:ti,ab,kw OR Drenthe:ti,ab,kw OR Gelderland:ti,ab,kw OR Flevoland:ti,ab,kw OR Limburg:ti,ab,kw OR Noord-Brabant:ti,ab,kw OR Overijssel:ti,ab,kw OR Utrecht:ti,ab,kw OR Belgium:ti,ab,kw OR Belgian:ti,ab,kw OR Belgians:ti,ab,kw OR Brussels:ti,ab,kw OR Flanders:ti,ab,kw OR Flemish:ti,ab,kw OR Flemings:ti,ab,kw OR Wallonia:ti,ab,kw OR Walloon:ti,ab,kw OR Walloons:ti,ab,kw OR Luxembourg*:ti,ab,kw)

AND

(epidemiology:lnk OR 'epidemiology'/exp OR epidemiol*:ti,ab,kw OR statistic*:ti,ab,kw OR incidence:ti,ab,kw OR rate:ti,ab,kw OR rates:ti,ab,kw OR distribution*:ti,ab,kw OR cases:ti,ab,kw OR prevalen*:ti,ab,kw OR 'gene frequency'/exp OR frequenc*:ti,ab,kw OR occurrenc*:ti,ab,kw OR 'observational study'/exp OR 'case control study'/exp OR 'retrospective study'/exp OR 'cohort analysis'/exp OR 'longitudinal study'/exp OR 'prospective study'/exp OR observational:ti,ab,kw OR case-control*:ti,ab,kw OR retrospective*:ti,ab,kw OR cohort*:ti,ab,kw OR longitudinal*:ti,ab,kw OR prospective*:ti,ab,kw OR cross-section*:ti,ab,kw OR transversal*:ti,ab,kw OR population-based:ti,ab,kw OR series:ti,ab,kw OR 'regression model'/exp OR regression:ti,ab,kw OR matching:ti,ab,kw OR matched:ti,ab,kw)
